# The effects of high-velocity hamstring muscle training on injury prevention in football players

**DOI:** 10.3389/fphys.2023.1219087

**Published:** 2023-08-21

**Authors:** Sigitas Kamandulis, Joan Aureli Cadefau, Audrius Snieckus, Mantas Mickevicius, Inga Lukonaitiene, Pornpimol Muanjai, Danguole Satkunskiene, Victor Molina, Xavier de Blas Foix, Daniele Conte

**Affiliations:** ^1^ Institute of Sports Science and Innovation, Lithuanian Sports University, Kaunas, Lithuania; ^2^ National Institute of Physical Education (INEFC), Barcelona University, Barcelona, Spain; ^3^ Department of Physical Therapy, Faculty of Allied Health Sciences, Burapha University, Chon Buri, Thailand; ^4^ Faculty of Psychology Education Sciences and Sport Blanquerna, Universitat Ramon Llull, Barcelona, Spain

**Keywords:** hamstring injury rate, injury prevention strategy, elastic band, leg dominance, training

## Abstract

**Background:** Explosive and fast body movements, sprints, jumps and quick changes of direction, which are characteristic of the football training, place considerable strain on the hamstring muscles. Due to the high occurrence of hamstring injuries, new preventive strategies are required that focus on high-velocity training. The purpose was to assess the effectiveness of high-velocity elastic-band training in reducing the occurrence of hamstring injuries in football players.

**Methods:** Male football players from 15 teams (n = 319) playing in national competitions participated in this study. The players were involved in a 5-week exercise period in either the intervention group (INT) or the control group (CON), with a follow-up period of ∼4 months where hamstring injuries and exposure time were recorded. The INT group had two to three sessions per week of elastic-band training with low-load, high-velocity leg curls while lying prone; the CON group performed self-paced football-specific drills.

**Results:** The incidence rate of hamstring injuries was 6.5% in the INT group (8 out of 123 players) and 9.2% in the CON group (18 out of 196 players). Although the INT group showed almost 1/3 reduction in hamstring injury incidence compared to the CON group, the difference was not statistically significant (*p* > 0.05). Moreover, no differences (*p* > 0.05, odds ratio [OR] = trivial-to-small) in distribution between the groups were found in hamstring injury characteristics (leg dominance and mechanism) except for the distribution of injuries that occurred during matches or training (*p* = 0.036; OR = 6.14, moderate).

**Conclusion:** The program of high-velocity elastic-band training did not prove to be effective in preventing hamstring muscle injuries in football players despite displaying some positive indications that could be considering when creating injury prevention programs.

## 1 Introduction

Hamstring muscle injuries are prevalent among football players, negatively affecting their performance and imposing significant financial burden on professional clubs (Hägglund et al., 2006; Ekstrand et al., 2013). Most hamstring injuries are non-contact in nature and occur during activities that involve rapid muscle contractions, such as sprinting, as well as when the muscles are stretched, such as when lunging, landing or performing high kicks (Woods et al., 2004; Askling et al., 2012; Gronwald et al., 2022). Despite advances in trauma treatment and rehabilitation, the risk of injury recurrence remains substantial (Green et al., 2020; Ekstrand et al., 2022). Therefore, characterization of hamstring injury prevention strategies seems fundamental for professional football players.

Resistance-type training involving concentric and eccentric movements is considered to be essential for reducing hamstring weakness and preventing musculoskeletal disorders (Mjølsnes et al., 2004; Croisier et al., 2008; Shalaj et al., 2020). Previous studies have highlighted the benefits of flywheel resistance (Askling et al., 2003) and Nordic hamstring exercise training (Al Attar et al., 2017; Raya-Gonzalez et al., 2021; Vatovec et al., 2021) in producing positive adaptations. However, these popular prevention strategies focus on developing maximal strength through exercises performed at low velocity, which may not be optimal for high-velocity movements. Notably, a significant proportion of hamstring injuries occur during high-velocity activities (Askling et al., 2012; Opar et al., 2012; Gronwald et al., 2022), indicating the need for new preventive interventions that specifically target high-velocity training.

The movement velocity elicited by elastic bands has been considered comparable to those attained during jumping or sprinting (Argus et al., 2011; Upton, 2011; Alcaraz et al., 2018). The use of elastic-band exercises with high-frequency knee flexion and extension at high velocity (lying prone curls) has been shown to produce hamstring strength improvement in non-athletes and basketball players (Janusevicius et al., 2017; Kamandulis et al., 2020). Overall, this training methodology entails an alteration in hamstring muscle recruitment during flexion–extension cycles, likely preventing excessive lengthening of the muscles during exercise (Kamandulis et al., 2020). These positive muscular and neural adaptations may well translate into a lower incidence of strain-type injuries in the hamstrings. Therefore, the aim of this research was to assess the effectiveness of high-velocity elastic-band training to reduce the occurrence of hamstring injuries in football players.

## 2 Materials and methods

### 2.1 Participants

Initially, over 650 eligible players (healthy and hamstring injury-free for the previous 12 months) from 30 professional and semi-professional football teams were enrolled. Because the study was conducted in both Lithuania and Spain, variations in methodology arose due to differences in championship schedules and environmental factors. Moreover, some Lithuanian teams were unable to adhere to the study’s design and withdrew from the experiment within championship autumn period. Therefore, only data collected during the Lithuanian championship spring period, with a total of 319 players from nine teams in the Lithuanian premier league and six teams in the Lithuanian 1st division, were used in the analysis. Players’ age, anthropometric characteristics, and dominant side are presented in Table 1. The study was approved by the Lithuanian Sports University Local Ethical Review Board in Kaunas, Lithuania, and was conducted in accordance with the principles of the Declaration of Helsinki.

**TABLE 1:** Age, anthropometrics and dominant side of football players in Intervention and Control groups (mean ± SD).

	Intervention	Control
Subjects, n	123	196
Age, years	24.6 ± 5.1	21.8 ± 4.5*
Stature, cm	181.9 ± 6.6	181.9 ± 6.7
Body mass, kg	75.9 ± 7.8	75.0 ± 8.2
Left dominant leg	17	24
Right dominant leg	106	172

**Note:** *p < 0.05, compared to Intervention group.

### 2.2 Design

The present investigation entailed the allocation of the recruited football teams to either an intervention group (INT), which underwent an elastic-band training program plus regular football training, or a control group (CON), which has performed self-paced football-specific drills in addition to regular football training. A stratified randomization method was used to balance their participation in similar level competitions, taking into account that teams in premier and first leagues have different training volume and game schedule intensity. The players were involved in a 5-week two to three times per week the INT or the CON group activities within January and February, specifically at the end of the preparation to national championship phase. This was followed by a follow-up period of up to 4 months (March-June) during the competition phase. Moreover, during the follow-up period, to maintain the training effects, players from the INT group repeated their specific exercise two to three times for 1 week every 4 weeks. Since the study was carried out during the COVID-19 pandemic, in the case where players became infected (17 cases), they were instructed to resume the program immediately after isolation period from where they left before becoming infected. The isolation period was not included in the analysis.

### 2.3 Hamstring exercise procedures

**FIGURE 1:**
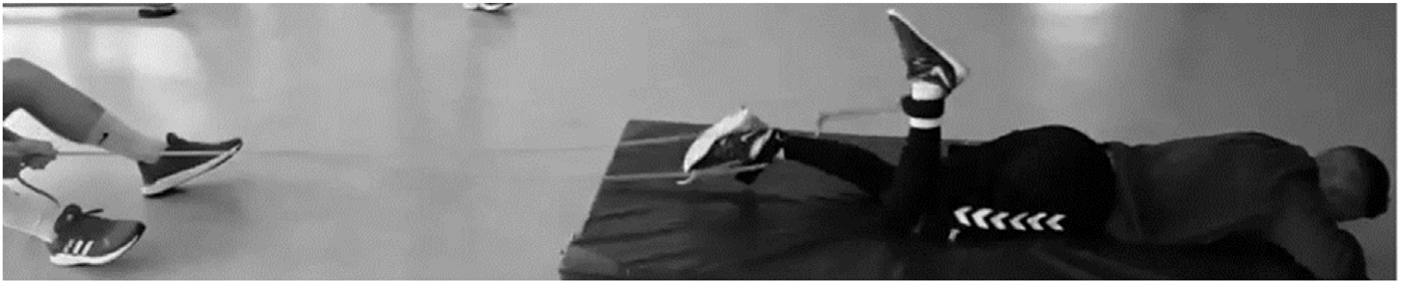
The hamstring curl exercise for the intervention group.

The hamstring curl exercises for the INT group were performed using TheraBand™ (Akron, OH, United States) silver rubber bands. This type of band is classified as possessing high resistance and low elasticity, resulting in a resistance force of 8.6 kg when elongated to 100%. Each participant was required to lie face down on a mattress with their legs straight. The band were attached to the ankles with adjustable straps made of nylon webbing and reinforced steel D-rings. Then, knee flexion and extension at the full range of motion from the ground to the buttocks was performed, alternating the legs as quickly as possible at maximal velocity for a total of 4 s (Figure 1). Exercises progressed from four sets in weeks one and two to five sets in weeks three and four to six sets in week six. The sets were interspersed by a 1-min rest. Participants exercised with a 1-m resistance band during the first week. Successively, more resistance was applied, extending the band by an extra 1 m during weeks two to three and once more during weeks four to five. Before reaching maximal intensity, warm-up repetitions without the use of the elastic bands were performed as follows: 50% intensity (10 s), 70% intensity (7 s), 90% intensity (4 s). The participants controlled intensity of the exercise by subjective effort perception.

The INT group activities were carried out immediately after general warm-up by the team conditioning coach or physiotherapist, who was familiarized with the training methods before the commencement of the study. Moreover, supplementary online video clips showing how to correctly do the exercises were provided for participants and team staff.

### 2.4 Injury and time exposure recording

Hamstring injuries were recorded according to the international consensus for epidemiological studies in professional football (Fuller et al., 2006) and were classified according to training or match incidence, leg dominance and mechanism. The duration between the day of injury and the day the player returned to full participation in training or match was utilized to quantify the severity of the injury (Soligard et al., 2008). The club physician, and in certain instances, the physiotherapist, assumed the role of the primary contact person responsible for collecting injury-related data, including the diagnosis. For this purpose, an online platform was created to record players’ injuries as well as the number of training sessions and number of weekly matches. The online platform also served to monitor players’ adherence to the intervention program, with compliance rates reaching 85%. No effect of the intervention was expected until full completion of the hamstring curl exercises protocol as having carried out minimum 12 training sessions. Therefore, hamstring injuries were recorded only during the follow-up periods. To avoid recording the same injury multiple times, the recurrence incidents of already recorded injuries was excluded.

### 2.5 Statistical analysis

Descriptive statistics (mean and standard deviation (SD)) were used to assess participants’ baseline characteristics and exposure data. Moreover, percentages were used as descriptive statistics for hamstring injury incidents. The Shapiro–Wilk test was used to check data distribution of baseline measurements revealing normal distribution for stature, while body mass and age were non-normally distributed. Therefore, the differences between INT and CON groups at baseline levels were assessed using the Mann–Whitney *U* test for age and body mass and using an independent *t*-test for stature after checking the assumption of equal variance assessed via Levene’s test.

Fisher’s exact test was adopted to assess the differences in distribution of injured and non-injured players between the INT and CON groups. Moreover, considering only players suffering hamstring injuries, Fisher’s exact test was adopted to assess the differences between INT and CON groups in 1) leg dominance (non-dominant vs. dominant); 2) injury mechanism (non-contact vs. contact); and 3) activity type (training vs. match). Odds ratios (ORs) with 95% confidence intervals (CI) were also calculated as effect sizes and interpreted according to Hopkins’ benchmark considering 1.0, 1.5, 3.5, 9 and 32 as trivial, small, moderate, large and very large effect sizes, respectively (Hopkins et al., 2009). Relative risk (RR) was calculated to compare the injury risks for the INT and CON groups.

Finally, differences in age, stature and body mass between injured and non-injured players were assessed by Mann–Whitney U test. An alpha level of *p* < 0.05 was set *a priori* for statistical significance. All data were analysed using Jamovi software (version 2.3.21.0, 2023).

## 3 Results

Baseline measurements showed no significant differences in body mass (*p* = 0.151) and stature (*p* = 0.998) between the INT and CON groups, but the INT group had higher average age (about 2.7 years, *p* < 0.001). Twenty-six hamstring injuries were recorded over the follow-up period across the two groups. The INT group reported 8 hamstring injuries in 123 players (6.5%), whereas the CON group reported 18 injuries in 196 players (9.2%). Although the relative risk was observed to be 29.0% lower in the group of individuals who underwent the intervention (RR = 0.71), as compared to the control group, the distribution of injuries between the INT and CON groups was not found to have a statistically significant difference (Table 2).

**TABLE 2:** Comparison in the distribution of injured and non-injured players between control and intervention group.

Injury status	Groups		*p*-value	OR (95%CI)	Interpretation
	Control	Intervention			
Injured	18	8	0.529	0.69 (0.29; 1.63)	Trivial
Not injured	178	115			

Note: OR, odds ratio; 95%CI, 95% Confidence Interval.

The INT group reported 8 hamstring injuries in 123 players (6.5%), whereas the CON group reported 18 injuries in 196 players (9.2%). 69.2% of all injuries were in the control group and 30.8% - in the intervention group. There was no significant difference in the distribution of injured players in the CON and the INT group (Table 2); however, the results show a positive effect of the hamstring curl exercises on preventing injury in the INT group. The odds for players in the INT group to receive injury was about 30% less (relative risk (RR) = 0.71) than in the CON.

Overall, the number of hamstring injuries was similar between matches and training sessions (14 and 12 injuries, respectively) but different when considering exposure time (2.2 and 0.38 per 1,000 h, match and training time, respectively). When considering the total number of hamstring injuries, the majority were non-contact (n = 23) and with moderate severity (n = 17) and with an average recovery time of 19.5 ± 10.9 days per player. Moreover, no statistically significant differences (*p* > 0.05) in the distribution between CON and INT groups were found across leg dominance and mechanism, with trivial ORs except for sport activity (*p* = 0.036; OR [95% CI] = 11.0 [1.1; 110.0], very large) (Table 3). Finally, players suffering hamstring injuries were similar in age (mean difference: 1.2 years; *p* = 0.067), stature (mean difference, 0.3 cm; *p* = 0.826) and body mass (mean difference, 0.8 kg; *p* = 0.425) compared with their non-injured counterparts.

**TABLE 3:** Comparison in the distribution of the injury characteristics between control and intervention group.

Injury characteristics		Groups		*p*-value	OR (95%CI)	Interpretation
		Control	Intervention			
Leg dominance	Non-Dominant	7	4	0.683	0.64 (0.12; 3.41)	Trivial
	Dominant	11	4			
Mechanism	Non-contact	16	7	1.000	0.87 (0.07; 11.3)	Trivial
	Contact	2	1			
Activity	Training	11	1	0.036	11.0 (1.10; 110.0)	Very Large
	Match	7	7			

Note: OR, odds ratio; 95% CI, 95% Confidence Interval.

## 4 Discussion

The aim of this study was to assess the effectiveness of high-velocity elastic-band training in reducing the occurrence of hamstring injuries in football players. The primary results indicate no significant variation in the incidence of hamstring injury between the intervention and control groups. Nevertheless, the suggested training approach, which incorporates high-velocity elastic-band exercises, was linked with a reduction of roughly 30% in the hamstring injury risk among professional and semi-professional football players, implying that this exercise type may have some potential protective value in the football settings. Further research on this topic is warranted to obtain a comprehensive understanding of its suitability for the hamstring trauma prevention.

Previous research has suggested that injury prevention strategies, including strength training using weight machines or Nordic hamstring exercises, may reduce hamstring injuries (Petersen et al., 2011; van Dyk et al., 2019), although Nordic hamstring exercises benefits extent has been recently questioned (Impellizzeri et al., 2021). While these prevention methods entail slow-motion exercises, neuromuscular adaptations to high velocities are obligatory, as other data suggest hamstring injuries frequently occur during high-velocity activities (Woods et al., 2004; Schache et al., 2012). Our results here report a reduction in hamstring injuries of about one-third following high-velocity elastic-band training, a reduction magnitude comparable to that previously reported for traditional interventions (Al Attar et al., 2017; van Dyk et al., 2019). Overall, the outcomes may still stand potential relevance, even statistical difference between the intervention group and the control group did not attain significance. It is imperative to acknowledge the limitations of utilizing a singular approach in preventing injuries, and to recognize that transient interventions may not fully mitigate risks that have accrued over an extended period with certain risk factors remaining unmodifiable. We also can speculate that the high-velocity elastic-band training induced an increase in knee flexion strength at high velocities, hamstring muscle activation during the flexion–extension cycles and cooperation of antagonistic muscles contractions, as has been suggested in a previous investigation (Kamandulis et al., 2020). Indeed, the specific mechanisms underlying these adaptations to high-velocity elastic-band training were not explicitly delineated in the current study.

From an epidemiological standpoint, our findings are in line with previous results assessing hamstring injury occurrence in football players (Ekstrand et al., 2016; López-Valenciano et al., 2020). Overall, we documented an injury rate of 8.1% among players within the 4-month follow-up in-season period with a rather long average recovery time (19.5 days) and a higher injury prevalence in matches compared with that in training sessions (about 6 times over the same exposure time). Additionally, more than half of the registered hamstring injuries occurred in players >24 years old, which overall represented only 31% of the total sample. This trend are in line with the previous body of literature indicating that age is a risk factor for hamstring injuries in football players (Henderson et al., 2010). It should also be noted that three of 26 (11.5%) players reported hamstring injuries at age 18–19 years, which confirms the absolute need to undertake prevention strategies on a daily basis, independent of players’ age.

From a practical standpoint, the use of hamstring training with knee flexions/extensions using elastic bands while lying prone has many beneficial aspects. Indeed, compared with more traditional exercises, it is safer because the face-down prone position allows the hamstring muscles to adapt to high-speed movements with the athlete not using their body weight. Moreover, it should be noted that the use of elastic bands creates a unique load distribution. In fact, elastic bands provide a proportionally increasing resistance on the hamstrings during the knee flexion phase, making hamstrings stronger concentrically. During the knee extension phase, the extensor muscles receive a proportionally increased amount of assistance increasing the quadriceps muscle group’s contraction velocity. As a result, the hamstring which represent the opposing muscle group are forced to adapt to the amplified velocity demands since they actively participate in slowing down and stopping the knee extension, making hamstring stronger eccentrically. It was shown previously that eccentric knee flexor strength reduces the risk of hamstring injury in elite football (Timmins et al., 2016). With elastic bands, athletes can reach almost the same velocity as they can without resistance, with no alteration of the exercise technique, and produce a high velocity, which is considered critical for this kind of training.

The study had a limitation in that the training and follow-up period experienced some interruptions due to the COVID-19 pandemic, with a small percentage of players testing positive for the virus (<6%) or receiving vaccination. None of the players required hospitalization. It seems this did not affect our results considering that a similar number of injuries were recorded, compared with previous investigations (Ekstrand et al., 2016; López-Valenciano et al., 2020). Another limitation pertains the enrolment of a considerable number of players who were under the age of 20, particularly in the control group. This may have resulted in a slight underestimation of the occurrence rate of hamstring injuries in the control group, as advancing age is considered an important non-modifiable risk factor in hamstring injuries (Opar et al., 2012; Freckleton and Pizzari, 2013; Green et al., 2020). Finally, we were unable to account for additional potential confounding factors such as player competency and team coaching efficiency or individual numbers of acceleration, cutting, and deceleration movements per player during a match since these aspects are extremely challenging to account for in applied settings.

The study has contributed valuable insights into the significance of muscle adaptation that occurs due to high-velocity training. It highlights that such adaptation could potentially enhance the effectiveness of hamstring injury prevention programs for football players. Nonetheless, the effectiveness of the training program that was developed could not be established. This could be attributed to suboptimal training design or the lack of a comprehensive training approach that considers the various factors that increase the risk of hamstring injuries. Thus, further investigation is necessary to determine the most suitable training combination for football strength and conditioning coaches.

## 5 Conclusion

The program of high-velocity elastic-band training, although demonstrating some encouraging indications that could have been considered in devising injury prevention programs, did not demonstrate effectiveness in preventing hamstring muscle injuries in football players. It would be worthwhile to search further for the optimal characteristics of high-velocity hamstring muscle training programs and establish their effects in combination with other hamstring strengthening methods.

## Data Availability

The raw data supporting the conclusion of this article will be made available by the authors, without undue reservation.
